# Rapid Processing of a Global Feature in the ON Visual Pathways of Behaving Monkeys

**DOI:** 10.3389/fnins.2017.00474

**Published:** 2017-08-25

**Authors:** Jun Huang, Yan Yang, Ke Zhou, Xudong Zhao, Quan Zhou, Hong Zhu, Yingshan Yang, Chunming Zhang, Yifeng Zhou, Wu Zhou

**Affiliations:** ^1^CAS Key Laboratory of Brain Function and Disease, and School of Life Sciences, University of Science and Technology of China Hefei, China; ^2^Department of Otolaryngology and Communicative Sciences, University of Mississippi Medical Center Jackson, MS, United States; ^3^State Key Laboratory of Brain and Cognitive Science, Institute of Biophysics, Chinese Academy of Sciences Beijing, China; ^4^College of Psychology and Sociology, Shenzhen University Shenzhen, China; ^5^Center for Language and Brain, Shenzhen Institute of Neuroscience Shenzhen, China; ^6^Shenzhen Key Laboratory of Affective and Social Cognitive Science, Shenzhen University Shenzhen, China; ^7^Department of Neurobiology and Anatomical Sciences, University of Mississippi Medical Center Jackson, MS, United States; ^8^Department of Neurology, University of Mississippi Medical Center Jackson, MS, United States; ^9^Primate Research Center of Jin Gang International Haikou, China; ^10^Department of Otolaryngology, First Affiliated Hospital, Shanxi Medical University Taiyuan, China

**Keywords:** topological perception, visual search, feature integration, saccade, behaving monkeys

## Abstract

Visual objects are recognized by their features. Whereas, some features are based on simple components (i.e., local features, such as orientation of line segments), some features are based on the whole object (i.e., global features, such as an object having a hole in it). Over the past five decades, behavioral, physiological, anatomical, and computational studies have established a general model of vision, which starts from extracting local features in the lower visual pathways followed by a feature integration process that extracts global features in the higher visual pathways. This local-to-global model is successful in providing a unified account for a vast sets of perception experiments, but it fails to account for a set of experiments showing human visual systems' superior sensitivity to global features. Understanding the neural mechanisms underlying the “global-first” process will offer critical insights into new models of vision. The goal of the present study was to establish a non-human primate model of rapid processing of global features for elucidating the neural mechanisms underlying differential processing of global and local features. Monkeys were trained to make a saccade to a target in the black background, which was different from the distractors (white circle) in color (e.g., red circle target), local features (e.g., white square target), a global feature (e.g., white ring with a hole target) or their combinations (e.g., red square target). Contrary to the predictions of the prevailing local-to-global model, we found that (1) detecting a distinction or a change in the global feature was faster than detecting a distinction or a change in color or local features; (2) detecting a distinction in color was facilitated by a distinction in the global feature, but not in the local features; and (3) detecting the hole was interfered by the local features of the hole (e.g., white ring with a squared hole). These results suggest that monkey ON visual systems have a subsystem that is more sensitive to distinctions in the global feature than local features. They also provide the behavioral constraints for identifying the underlying neural substrates.

## Introduction

Extensive investigations over the past five decades have led to a general model of visual information processing, which starts from extracting local features of the retinal images in the lower visual pathways followed by integrating the local features to extract global features in the higher visual pathways (Hubel, 1988). Consistent with this model, neurons in the lateral geniculate nucleus (LGN) exhibit fine center-surround receptive fields that support extracting edges in the retinal images (Hubel, 1988; Van Hooser et al., 2013); neurons in the primary visual cortex (V1) exhibit elongated receptive fields that support extracting orientation of line segments (Hubel and Wiesel, 1962, 1963, 1977; Hubel, 1982; Tootell et al., 1998; Yacoub et al., 2008); neurons in the inferior temporal cortex, however, exhibit large receptive fields that support extracting more complex features of objects, such as hands and faces (Gross et al., 1972; Bruce et al., 1981; Bruce, 1982; Schwartz et al., 1983; Desimone, 1991). Behavioral studies provided further evidence to support the early feature detection and later feature integration theory. Using a visual search paradigm, Treisman and Gelade (1980) found that primitive features, such as color or orientation of line segments are extracted effortlessly, and in parallel over the entire visual field, and registered in special modules of feature maps. They suggested that in a later stage, focal attention is required to recombine the separate features for object recognition. Motivated by the anatomical, physiological and behavioral studies of visual systems, Marr (1982) proposed an influential computational system of vision, in which the simple components, and their geometric properties, typically as line segments with slopes, are used as representations of the retinal image in the early stages of its analysis. Over the past half century, the local-to-global theory of visual information processing has gained general acceptance and dominates the field of vision research.

Contrary to the early feature analysis approach, there is an early holistic registration approach, which suggests that perceptual processing is from global to local, i.e., wholes are coded prior to perceptual analysis of their separable properties or parts. Despite the general acceptance of the local-to-global approach, there is accumulating evidence for the global-to-local approach, including configural superiority (Weisstein and Harris, 1974; Navon, 1977), early detection of topological properties (Chen, 1982, 1990; Chen and Zhou, 1997), topological perception and long-range apparent motion (Chen, 1985; Zhuo et al., 2003; Wang et al., 2007; Zhou et al., 2010), detection of topological properties in bees (Chen et al., 2003), invariant shape processing in rats (see review, Zoccolan, 2015), visual perceptual learning (Ahissar and Hochstein, 2004), and holistic detection of Mooney faces, and objects (Bona et al., 2016). In particular, Chen (1982) and Chen et al. (2003) showed in humans and bees that two shapes (e.g., circle and ring) that were different in the hole feature (a global feature) were more discriminable than other pairs of shapes that were different in local features (e.g., circle and square). Nonetheless, the neural substrates underlying the global-to-local processes remain elusive, and there is a big knowledge gap in reconciling the local-to-global, and the global-to-local approaches. The main goal of the present study is to test the hypothesis that monkey visual systems rapidly detect the hole feature over local features. If successful, it not only adds support to the “global-first” theory, but also provides a robust platform for elucidating the neural mechanisms of processing global and local features by neurophysiological, and neuropharmacological approaches. This study was also motivated to reconcile the inconsistent reports in the limited monkey literature on processing the hole feature. While Komatasu and Ideura (1993) showed that a group of neurons in the monkey inferior temporal cortex were selectively activated by shapes with a hole, regardless of the shape of the holes, Shen et al. (2002) showed that hole precedence in monkeys was not in shape discrimination, but limited to face discrimination. In this study, we employed the pop-up visual search paradigm (Treisman and Gelade, 1980; Bichot and Schall, 1999) to directly compare the latencies to detect the targets that were different from the distractors in local features, global features, and color. Our results showed that monkey visual systems rapidly detected the hole feature, consistent with the human literature. Critical assessment of the local-to-global and global-to-local models will not only address the controversial but fundamental question of vision, i.e., what are the primitives of vision, but also provide important insights into cortical, and subcortical visual mechanisms in health and diseases.

## Methods

Two macaque monkeys (M1 and M2) were fitted to a head positioning platform. Eye movements were monitored by a magnetic search coil technique (Robinson, 1963; Judge et al., 1980; Zhou and King, 1998). Surgical procedures and experimental protocols were approved by the Institutional Animal Care and Use Committee at University of Mississippi Medical Center.

### Visual stimuli and eye movement measurement

The visual stimuli were a set of geometric shapes (2° in size) arranged with equal distances (4°) to the central fixation point. In the set, one object was the target, the others were distractors, i.e., white circles. The target was different from the distractors in color (e.g., red circle), local features (e.g., white square), a global feature (e.g., white ring with a black hole) or their combinations (e.g., red ring). The visual stimuli were displayed on a SAMSUNG PN64F5300AF Plasma TV (PN64F5300) monitor driven by an NVIDIA GT 610 video card. The monitor was located at 140 cm in front of the monkey. It had a display area of 140 × 80 cm, with a resolution of 1,920 × 1,080 pixels. The refresh rate of the monitor was 60 Hz. Using a Digital Lux meter LX1330B, the monitor background luminance was measured as 0.2 cd/m^2^, the luminance of the white, red, green and blue surface was measured as 270, 43, 155, and 76 cd/m^2^, respectively. It is important to note that early topological perception studies in humans (Chen, 1982, 1990) presented black stimuli against a white background, therefore, demonstrating detection of topological properties in human OFF visual pathways. In this study, the stimuli were presented against a black background and primarily activated monkey ON visual pathways. However, Yeh et al. (2009) showed that “black” responses dominate monkey V1 and later visual cortical areas. Thus, it is important to limit our conclusions to the ON visual pathways and further investigate how the monkey OFF visual pathways detect global features in future studies. Stimulus onset was determined by a photocell positioned in the upper left corner of the monitor. Monkeys viewed the stimuli binocularly and were trained to make saccades to fixate the target for juice rewards. Eye positions were calibrated by requiring the monkeys to fixate spots at known horizontal and vertical eccentricities.

### Experimental conditions

**Figure 1:**
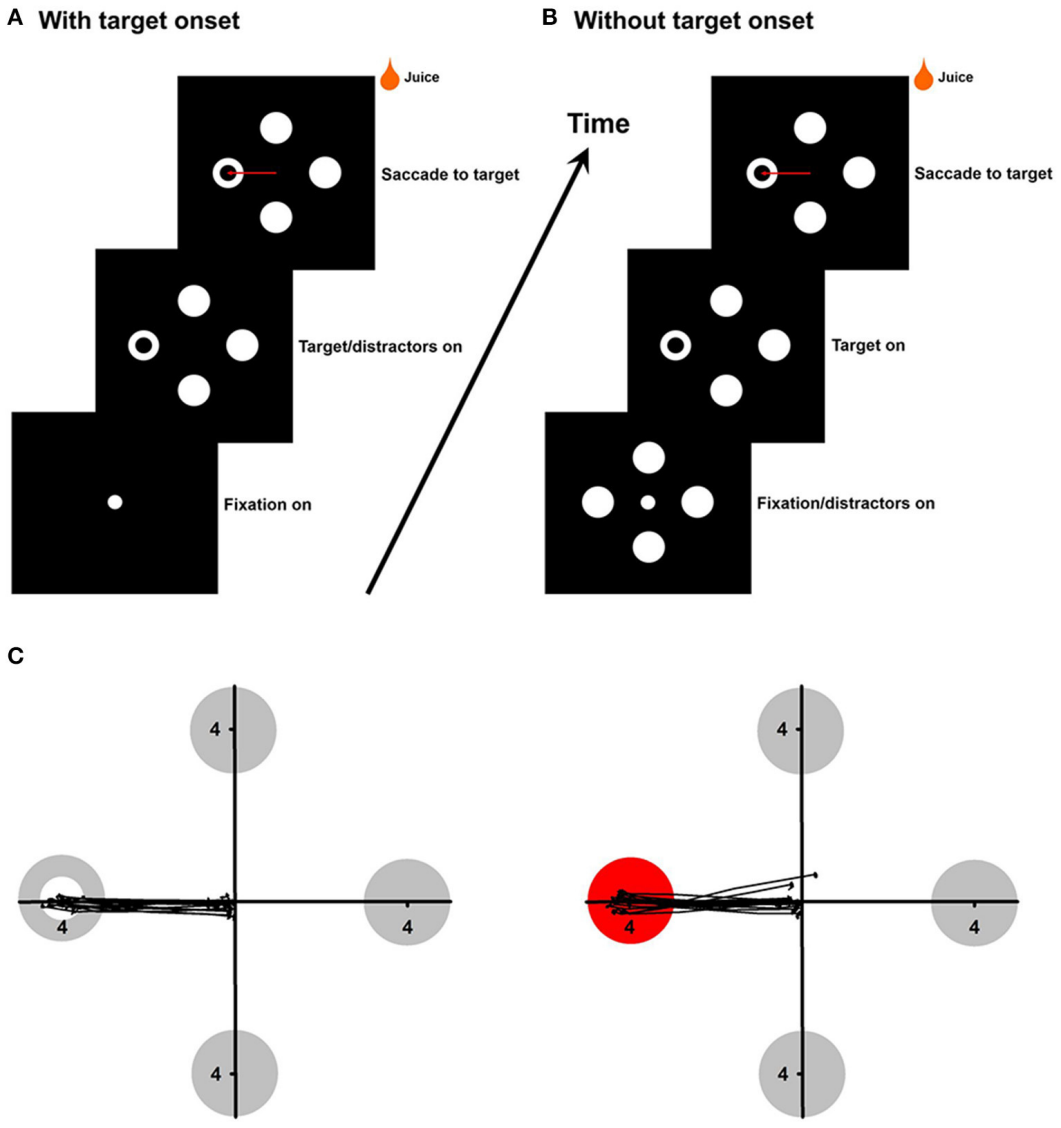
**(A,B)**, Experimental paradigms in the with-onset and without-onset conditions. **(C)** Saccade trajectories to the ring target (left panel) and the red circle target (right panel). Each black line represents one saccade trajectory.

In the first experiment, the target and distractors appeared simultaneously after the fixation point (500–700 ms) was extinguished (Figure 1A). The target appeared randomly in one of the four or eight locations (four cardinal and four oblique directions) at eccentricities 4°. The target was different from distractors in color (red/green/yellow/blue circle vs. white circles), local features (white square vs. white circles), area (white ring target with different hole sizes or small white circle target with different diameters) or the global feature (white ring vs. white circles) (Figure 1C). In the second experiment, the distractors were presented first and after a fixation hold, one of the distractors was changed to the target while the central fixation point was turned off (Figure 1B). Unlike the first experiment, in which target onset was present in each trial, this experiment allowed us to selectively assess detecting a change in color, local features or the global feature. In the third experiment, the target was different from the distractors not only in color, but also in local features or the global feature. For example, the monkeys were asked to detect a red square from white circles, or a red ring from white circles. In the fourth experiment, the target was different from the distractors in both the global feature and local features. For example, the monkeys were asked to detect a white ring with a square-shape hole from white circles, or a white ring with a square-shape outline from white circles.

### Data acquisition and data analysis

**Figure 2:**
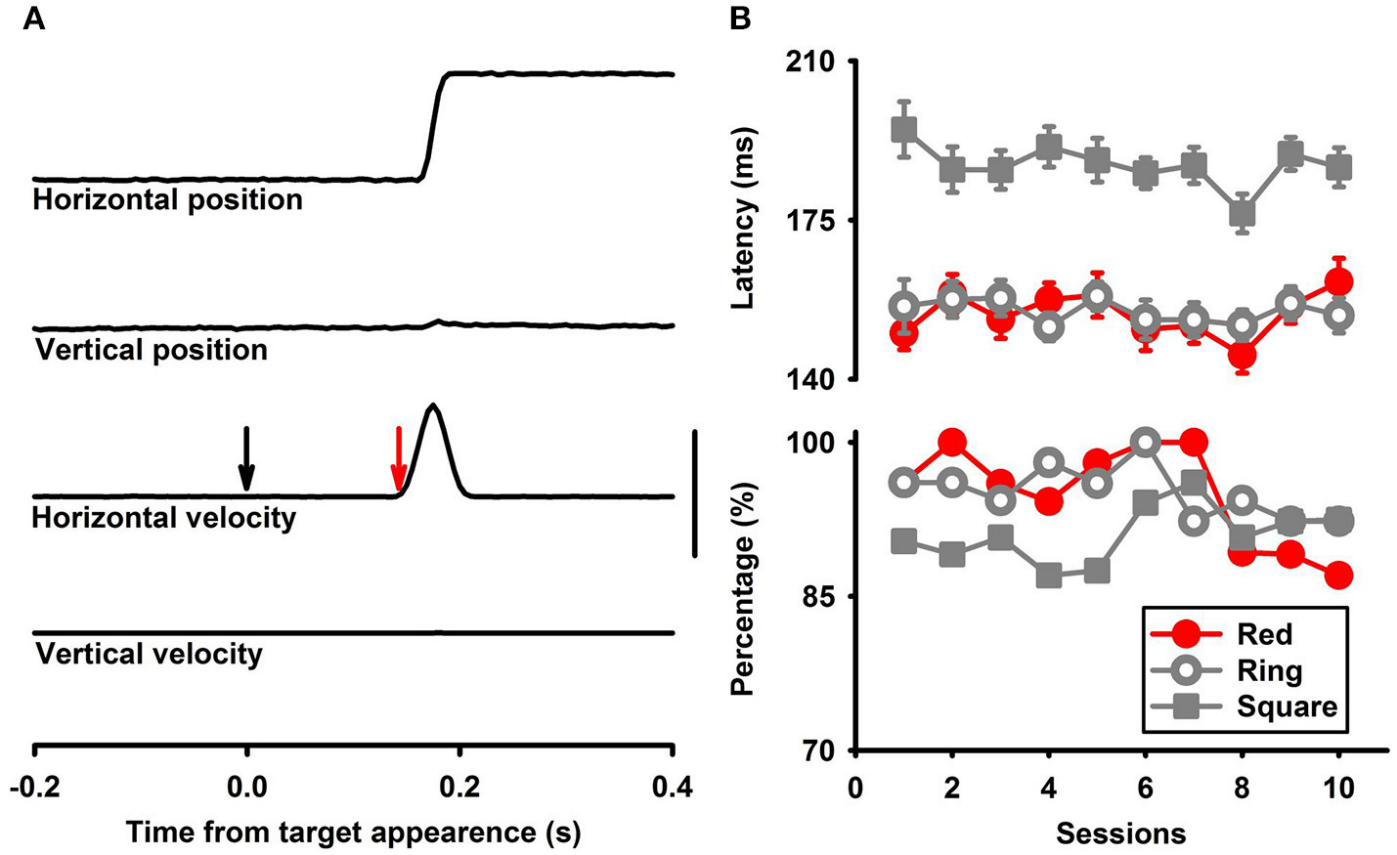
**(A)** Horizontal and vertical eye position and velocity signals. The black arrow indicates target onset, and the red arrow indicates saccade onset. The vertical bar is 4° for eye position and 150° for eye velocity. **(B)** Representative saccade latencies (upper panel) and accurate rates (lower panel) in multiple sessions for M1.

The experiments were controlled by BESC (High Tech Serv, Rochester, NY), in which experimental conditions were formulated as temporal sequences of instructions to operate devices (turn on and off visual stimuli, check fixation, deliver a drop of juice, etc.). A CED Power 1401 system (Cambridge Electronics Devices, Cambridge, UK) was used for data acquisition. Signals of eye coils and photocells were sampled at 1 kHz with 16-bit resolution. These signals were stored on a hard disk for offline analysis. Eye movement responses were analyzed using Spike2 (Cambridge Electronics Devices, Cambridge, UK), Matlab (Mathworks, Natick, MA) and SigmaPlot (Systat Software, San Jose, CA). Raw eye position data were filtered and differentiated with a band-pass of DC to 100 Hz to obtain eye velocity data. Trials in which monkeys made a saccade within 50 ms of the onset of the visual stimuli were rejected. Saccade onset was determined as the first point in the velocity trace over 20 deg/s (Figure 2A). The latency averages and percentage averages of correct trials were computed based on 250 trials in each condition (usually 5 sessions, 50 trials per session) (Figure 2B) (Bronstein and Kennard, 1987; Kalesnykas and Hallet, 1987; Smith and Van Gisbergen, 1989; Wenban-Smith and Findlay, 1991; Fischer and Weber, 1993; Fischer et al., 1993; Pare and Munoz, 1996; Zhou and King, 2002). ANOVA, paired *t*-test and *post hoc* comparisons were used to assess significance of the main effects and differences between conditions.

## Results

### Rapid detection of the ring target

**Figure 3:**
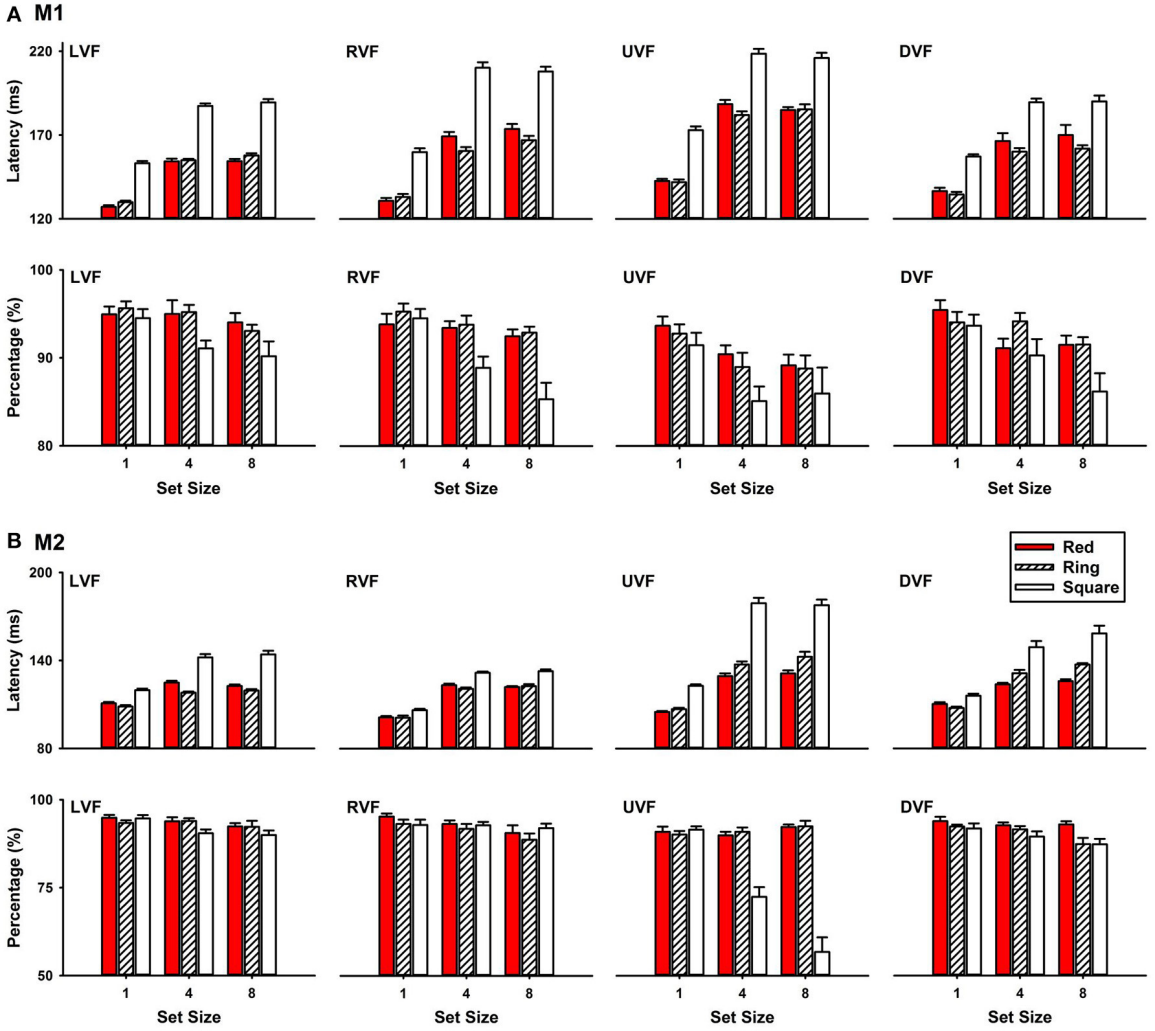
Saccade latencies to targets at eccentricity 4° in the left, right, upper and down visual fields (LVF, RVF, UVF, and DVF) **(A)**, M1; **(B)**, M2. All percentages of correct trials were large than 80%.

Figure 3 shows that saccade latencies to the white ring target were shorter than that to the white square target, but similar to that to the red circle target for the two monkeys (M1 and M2). During the experiments, the target appeared randomly in one of the four or eight locations of the stimulus set. For clarity, our analysis was focused on the trials with the target at the two horizontal locations. Since set size (4 or 8) had no effect on saccade latencies (M1: *P* > 0.05; M2: *P* > 0.05), data analysis was focused on the results in set size 4 condition. ANOVA showed that the differences among saccade latencies to the three targets were significant (*P* < 0.001). *Post-hoc* comparisons further show that saccade latencies to the ring target were shorter than that to the square target (M1: *P* < 0.001; M2: *P* < 0.001), but similar to that to the red circle target (M1: *P* > 0.05; M2: *P* > 0.05). For M1, detection of the ring target in the left visual field (LVF) had a latency of 155.01 ± 0.86 ms, which was not different from the latencies to detect the red circle target (154.39 ± 1.64 ms, *P* = 0.744), but was significantly shorter than the latencies to detect the square target (187.13 ± 1.58 ms, *P* < 0.001) (Figure 3A, LVF). For M2, saccade latency to the ring target (118.03 ± 0.81 ms) was similar to that to the red target (124.87 ± 1.32 ms; *P* > 0.05), but shorter than that to the square target (142.11 ± 2.32 ms; *P* < 0.001) (Figure 3B, LVF). Both monkeys correctly detected the targets in over 90% of the trials in LVF (M1: 93.7%; M2: 92.9%). Similar results were obtained for targets in the right visual field (RVF).

**Figure 4:**
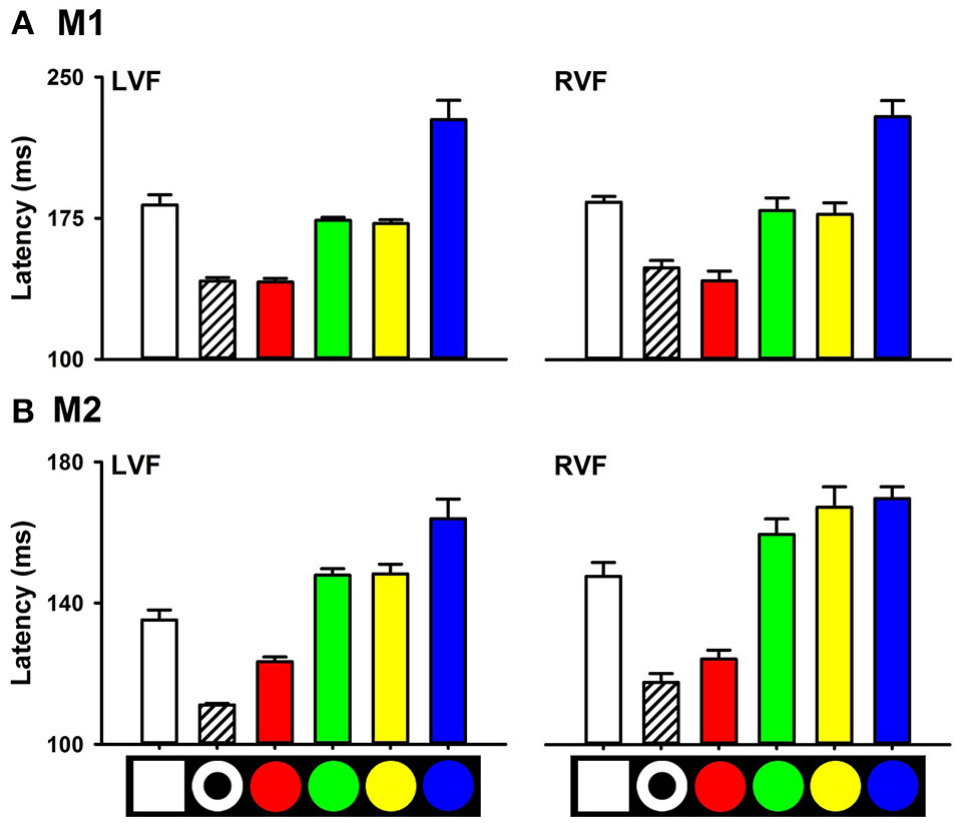
Saccade latencies to targets with different colors **(A)**, M1; **(B)**, M2.

We also measured saccade latencies to targets with other colors, such as blue, green, and yellow. ANOVA showed that there was a significant main effect of color on saccade latency. For M1, saccade latencies to the red circle target in the LVF were 141.15 ± 2.11 ms, which were shorter than that to the yellow (172.24 ± 2.19 ms, *P* < 0.001), green (173.96 ± 1.72 ms, *P* < 0.001) and blue (227.36 ± 11.42 ms, *P* < 0.001) circle targets. Similarly, for M2, saccade latencies to the red circle target (123.42 ± 1.48 ms) were shorter than that to the yellow (148.25 ± 3.06 ms, *P* < 0.001), green (147.90 ± 2.03 ms, *P* < 0.001) and blue (163.90 ± 6.20 ms, *P* < 0.001) circles (Figure 4). Similar results were obtained for targets with different colors in the RVF.

**Figure 5:**
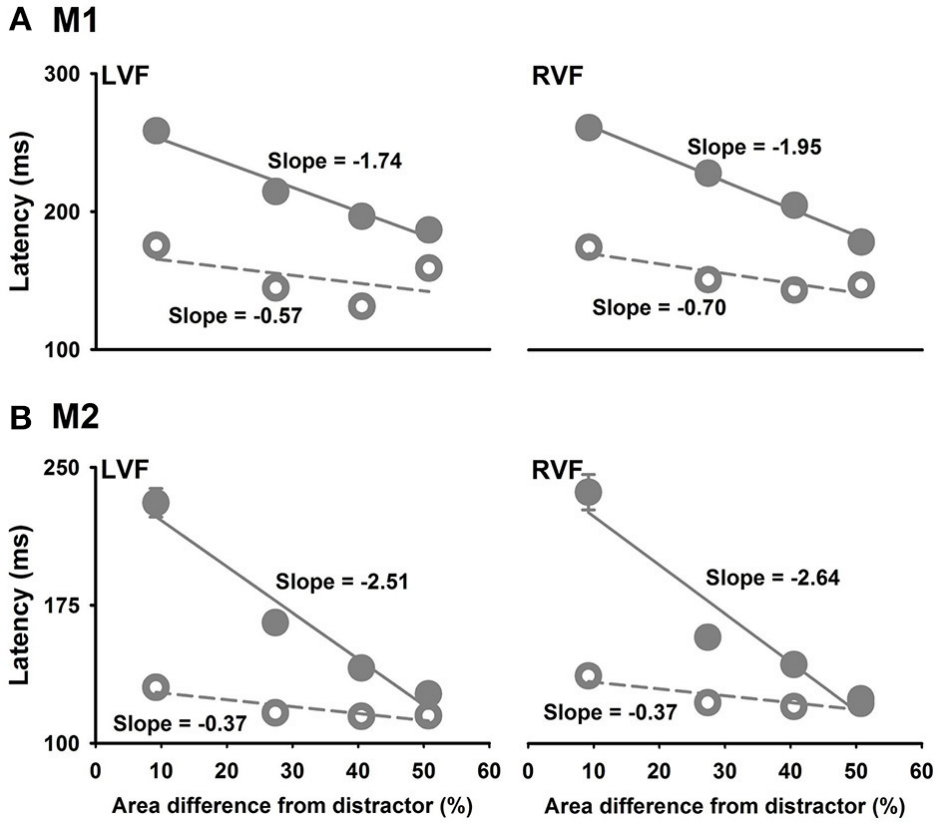
Saccade latencies as a function of the area difference between the target and the distractor. Filled symbols are for the smaller circle target and the open symbols are for the ring target. The unit of slope is ms per 1% **(A)**, M1; **(B)**, M2.

We further examined the effects of the size of the hole in the ring target on detecting the ring target by comparing saccade latencies to the ring targets with different hole sizes to saccade latencies to the smaller circle targets with similar area differences from the distractors. Linear regressions showed that saccade latencies to the smaller circle targets were dependent on the area differences between the target and the distractors with a slope of −1.74 ms per 1% area difference for M1 (LVF, *P* = 0.0191) and −2.51 ms per 1% area difference for M2 (LVF, *P* = 0.0219). However, saccade latencies to the ring target were independent of the area differences between the target and the distractors because the slopes were not significant from 0 for both monkeys (M1: −0.57 ms per 1% area difference, *P* = 0.4649; M2: −0.37 ms per 1% area difference, *P* = 0.1111) (Figure 5). Similar results were obtained for targets in the RVF of two monkeys. Note that the latency to the smaller circle targets were similar to that to the ring when the area difference from distractor was 50%.

### Rapid detection of a change in the global feature

**Figure 6:**
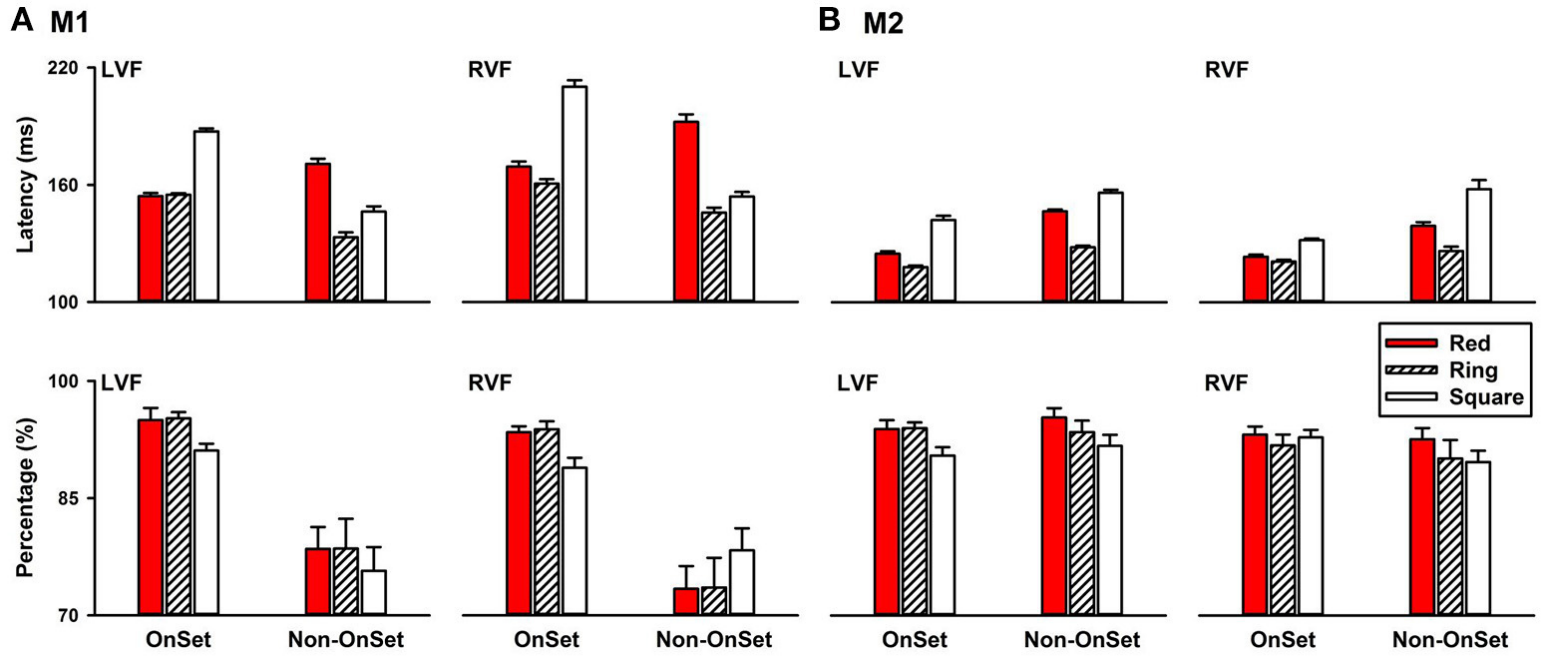
Saccade latencies of detecting a change in color, local features and the global feature in the left, right, upper, and down visual fields **(A)**, M1; **(B)**, M2.

Different from the above experiments, in which the target and the distractors were presented simultaneously, in this experiment, the distractor set were presented first followed by replacing one of the distractors with the target. An important difference between the two conditions was that stimulus onset was present in the first condition, but absent in the second condition. Without the stimulus onset, this paradigm allowed us to compare how visual systems detect a change in color, local features, and the global feature. Different from the conditions with target onset, in which detecting the ring was similar to detecting the red circle, in the without target-onset condition, detecting a change in the global feature was faster than detecting a change in color, and local features. For example, in the LVF, saccade latencies of detecting a change in the global feature (M1: 133.18 ± 2.77 ms; M2: 128.18 ± 0.89 ms) were shorter than that of detecting a change in color (M1: 170.74 ± 3.01 ms, *P* < 0.001, Figure 6A, LVF; M2: 146.58 ± 1.05 ms, *P* < 0.001; Figure 6B, LVF) in both monkeys.

### Effects of distinctions in local and global features on detecting the distinction in color

**Figure 7:**
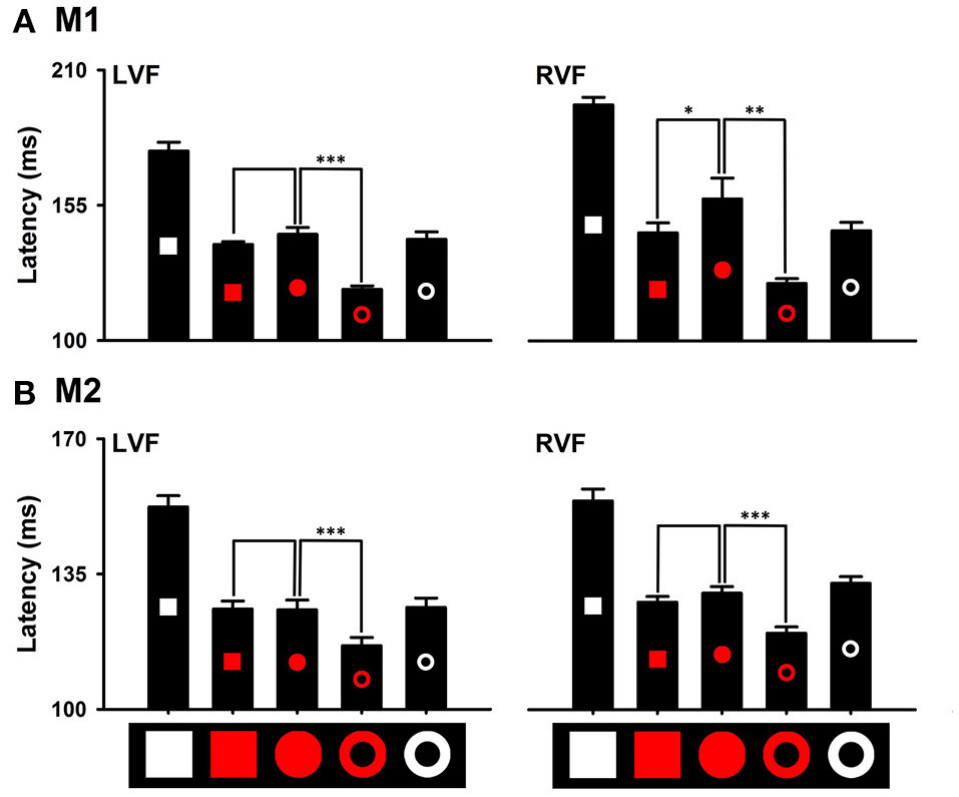
Effects of distinctions in the global and local features on detecting the distinction in red color **(A)**, M1; **(B)**, M2. ^*^P < 0.05; ^**^P < 0.01; ^***^P < 0.001.

**Figure 8:**
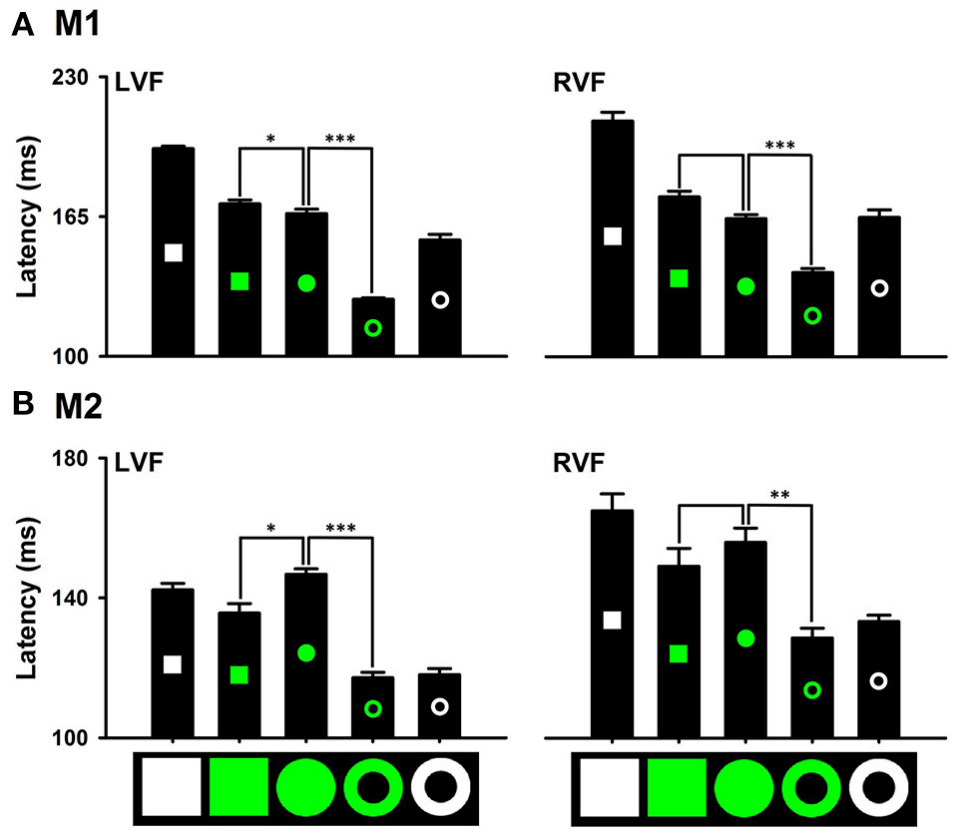
Effects of distinctions in the global and local features on detecting the distinction in green color **(A)**, M1; **(B)**, M2. ^*^P < 0.05; ^**^P < 0.01; ^***^P < 0.001.

Distinctions in local and global features exhibited differential effects on detecting the red target from the white distractors. Saccade latencies to the red ring target (M1: 120.85 ± 1.57 ms; M2: 116.47 ± 2.38 ms) were significantly shorter than that to the red circle target (M1: 143.24 ± 3.07 ms, *P* < 0.001; M2: 125.73 ± 2.83 ms, *P* < 0.001). However, saccade latencies to the red square target (M1: 139.12 ± 1.19 ms, *P* = 0.155; M2: 125.93 ± 2.30 ms, *P* = 0.848) were not different from that to the red circle target (Figure 7 LVF). Thus, the distinction in the global feature (i.e., ring) facilitates detecting the distinction in color, but the distinction in local features (i.e., square) does not have the facilitating effect. Similar results were obtained for detecting the other colors. For example, the distinction in the global feature facilitated detecting the green ring target in both monkeys [M1: 126.53 ± 0.72 ms (Green ring) vs. 166.43 ± 2.28 ms (Green disk), *P* < 0.001; M2: 117.19 ± 1.73 ms (Green ring) vs. 146.73 ± 1.74 ms (Green disk), *P* < 0.001], but the distinction in local features slowed down detecting the green square target in one monkey (M1: 170.98 ± 1.99 ms, *P* = 0.026) and marginally facilitated in the other monkey (M2: 135.62 ± 3.05 ms, *P* = 0.020) (Figure 8). When interpreting rapid detection of red color target, it is important to rule out luminance as a confounding factor. While the luminance of the red circle target (43 cd/m2) was lower than that of the white square target (270 cd/m2), the blue circle target (76 cd/m2) and the green circle target (155 cd/m2), detection of the red circle target was faster than that to the other targets. Thus, even though we did not use equiluminant colors, the results indicated that the effects of color could not be accounted for by differences in luminance.

### Effects of distinctions in local features on detecting the distinction in the global feature

**Figure 9:**
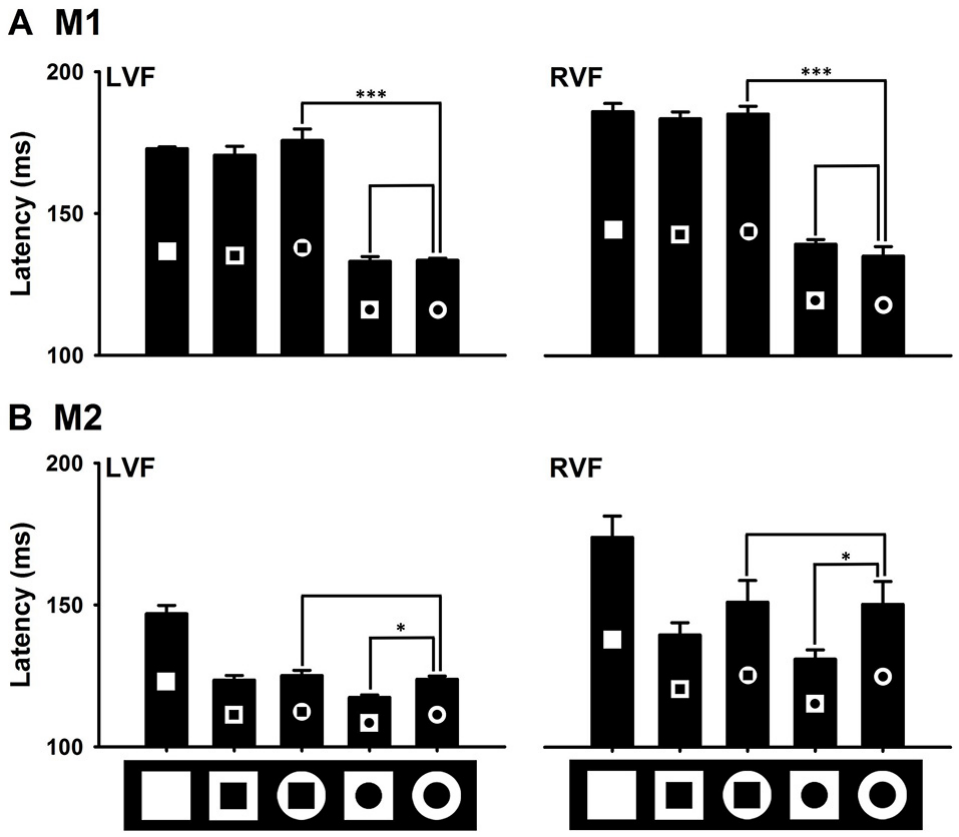
Effects of the distinction in local features on detecting the distinction in the global feature. Local features of the outside shape of the ring did not facilitate the detection of the ring in M1, but did it in M2 **(B)**. Local features of the shape of the hole in the ring slowed the detection of the ring target in M1 **(A)**, but did not in M2 **(B)**. ^*^P < 0.05; ^***^P < 0.001.

In this experiment, we examined effects of local features of the outside/inside shape of the ring on detecting the ring from the distractors (Figure 9). Interestingly, the two monkeys exhibited different effects. Compared to the standard ring with inside/outside circles, changing to the outside-square ring facilitated its detection for M2, but had no effect for M1. Changing to the inside-square ring slowed its detection for M1, but had no effect for M2. In other words, local features of the outside shape of the ring could facilitate ring's detection, but local features of the inside shape of the ring could slow ring's detection.

## Discussion

**Figure 10:**
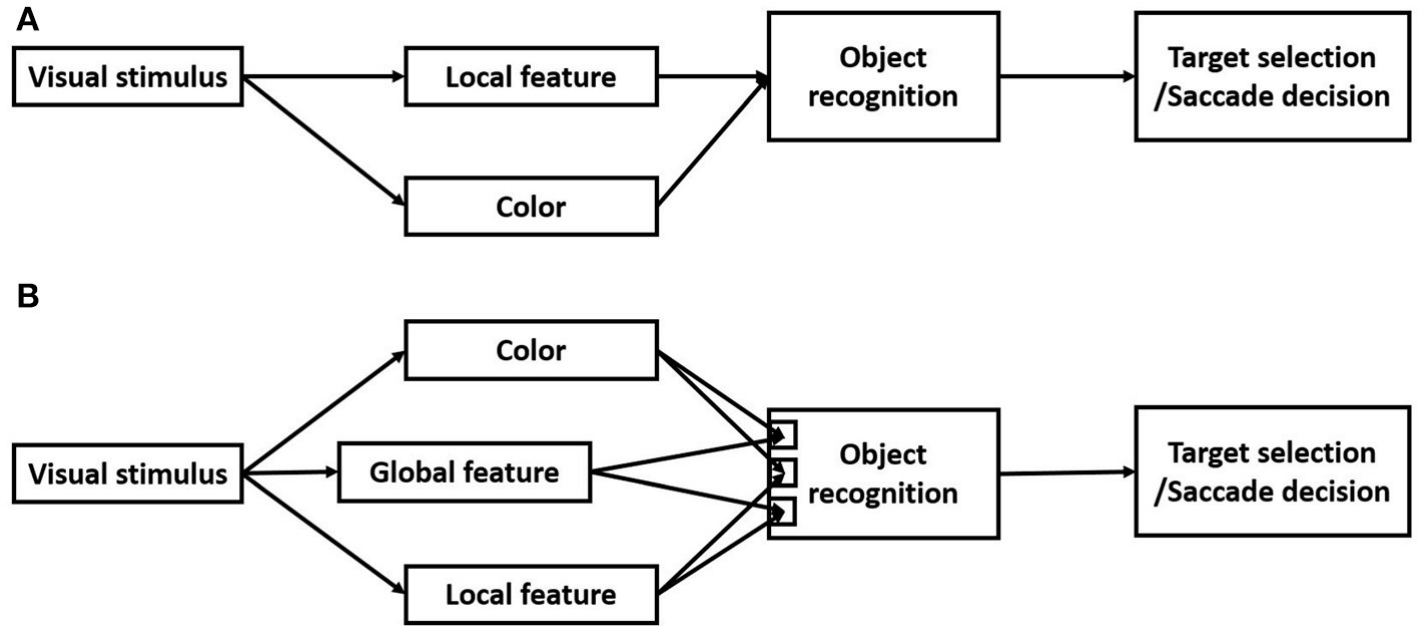
**(A)** Current local-to-global models of vision. Local features and color information are extracted in parallel in the early visual pathways. The global features are extracted in later visual pathways for object recognition. **(B)** A new model of vision based on the results of the present study. The global features are processed as a basic visual modality, and in parallel or even prior to the local features and color. However, there are interactions of processing the local features and the global features.

In this study, we employed the search detection task to investigate how monkey visual systems process global and local features. Whether a visual object has a hole in it was used as a representative global feature and line segments were used as representative local features. This study was motivated to test a seemly simple prediction of current models of vision. Since the local features are believed to be extracted in the early visual pathways and the global features are extracted in the later visual pathways by integrating the local features (Figure 10A), it is predicted that detecting distinctions or a change in local features takes less time than detecting distinctions or a change in the global feature. Contrary to this prediction, we found that detecting a distinction in the global feature took less time than detecting distinctions in local features (Figures 3, 6). We further found that detecting a change in the global feature was not only faster than detecting a change in local features, but also faster than detecting a change in color.

### Global feature as a basic visual modality

Previous studies have demonstrated human visual system early processing of global features characterized as topological invariants, such as whether a visual object has a hole (Chen, 1982, 2005a,b). However, the neural mechanisms underlying processing the global features remain to be elucidated. A critical obstacle is the lack of a non-human primate model of rapid detecting global features for neurophysiological investigations. To the best of our knowledge, this was the first study in behaving monkeys demonstrating rapid processing of a global feature, therefore, filling the important gap, and providing a valuable primate model system for future studies. The behavioral results further put important constraints on models of processing the global and local features and their interactions. For example, is the global feature extracted based on its local features, or as a basic visual modality? The results of this study suggested that the global feature “the object has a hole” is processed as a basic visual modality, likely in parallel with other basic visual modalities, such as local features, color, motion, and binocular disparity (Figure 10). This conclusion is based on the following results. First, detecting the global feature took less time than detecting the local features, therefore, it was unlikely based on integrating local features. Second, as shown in Figure 5, saccade latencies of detecting a smaller circle target was dependent on the area difference between the target and the distractor, but detecting the ring target was not affected by its area difference with the distractor. Therefore, rapid detection of the ring was not due to its area difference to the distractor, but due to the distinction in the global feature, i.e., the ring had a hole but the circle distractor did not. Third, detecting a change in the global feature was not only faster than detecting a change in local features, but also faster than detecting a change in color. Taken together, these behavioral results suggest that processing the global feature, i.e., number of holes in a visual object, is not based on processing local features, but in parallel or even prior to it.

### The superior colliculus (SC) as a potential site for rapid processing of the global feature

It has been well-established that the local features are first processed by the primary visual cortex, which receives retinal inputs via the lateral geniculate body pathways, and then integrated by the visual areas along the dorsal pathways, such as V2, V4, and inferior temporal cortex, which have neurons tuning to more complicated visual features, e.g., face and hand (Van Essen and Maunsell, 1983; Livingston and Hubel, 1988). However, little is known about where in the visual systems the global feature is processed. In fact, there is no study that directly examines the neural substrates that process the global features, such as whether an object has a hole. We suggest that the global feature is unlikely to be processed by the primary visual cortex because the global feature is directly extracted from the whole object, rather than by integrating its simple components, such as line segments. The global feature may be at least preliminarily processed by the superior colliculus (SC), which receives substantial direct retinal inputs and project to the cortical visual areas via the pulvinar nucleus of the thalamus (Pessoa and Adolphs, 2010). In the past several decades, visual responses of SC neurons have been extensively studied, but little is known on what the specific visual features are extracted by the SC neurons.

There are several lines of evidence suggesting a role of the SC in processing the global features. First, the SC neurons respond to the onset of visual targets without specific requirements for its local features. In other words, the SC neurons encode whether there is a new object in the scene, which is a global feature. Second, as shown by the seminal studies of Basso and Wurtz (1997, 1998), the visual responses of the SC neurons were modulated by the number of objects in the visual display or target uncertainty. Although the authors showed that the SC neurons did encode target uncertainty defined by factors other than visual configuration information, it is important to note that the SC neurons did encode the number of potential targets in each trial. Again, similar to target onset, which indicates the appearance of a new object in the scene, number of objects in the scene is also a global feature independent of local features. Since the latency of the target uncertainty effect was very short, nearly at the onset of visual responses of SC neurons, it is possible that number of visual objects in the display is processed within the SC circuits, without the need of cortical feedbacks. Third, neurons in the SC and V1 exhibit strikingly different responses to retinal image motion caused by the movement of an object in the world or of the eye itself across the visual scene. Robinson and Wurtz (1976) showed that most of the SC neurons differentiated external stimulus movement from image movement resulting from a monkey's own eye movement. Thus, the SC neurons would provide an uncontaminated visual signal to their target areas to facilitate decision making and sensorimotor transformation. In contrast to SC neurons, however, Wurtz (1969) showed that the V1 neurons did not discriminate between the stimulus movement and eye movement conditions. Fourth, the behavioral results showed that a distinction in local features did not speed up detecting a distinction in color, but a distinction in the global feature did. This finding is consistent with the study of White et al. (2009), which showed that the SC neurons effectively processed color information.

In summary, this study achieved two goals. First, we established a non-human primate model of rapid detection of a global feature of a visual object, i.e., whether it has a hole. This model lay the foundation for studying the neural mechanisms underlying differential processing of global and local features for visual object recognition. Second, the results put important constraints on future models of vision, which should treat the global feature as a basic visual modality like local features. It is worth noting that detecting local features and the global feature exhibited non-linear interactions. In particular, the local features of the hole of the ring slowed down detection of the hole, indicating possible interactions between the primary visual areas and the SC. Thus, new models should allow interactions between the modules of detecting global features and the modules of detecting local features in order to generate the observed behaviors. Future studies will further investigate a role of the subcortical pathways in processing global features in behaving monkeys. The new information will not only fill the current knowledge gap, but also provide constraints on new neural models of visual information processing.

## Author contributions

Study conception and design: WZ, YZ, JH, YaY, KZ. Acquisition of data: JH, YaY, XZ, QZ, HZ, YiY, CZ. Analysis and interpretation of data: WZ, YZ, JH, YaY, KZ. Drafting of manuscript: WZ, YZ, JH, YaY and KZ.

### Conflict of interest statement

The authors declare that the research was conducted in the absence of any commercial or financial relationships that could be construed as a potential conflict of interest.
